# IgA tetramerization improves target breadth but not peak potency of functionality of anti-influenza virus broadly neutralizing antibody

**DOI:** 10.1371/journal.ppat.1007427

**Published:** 2019-01-03

**Authors:** Shinji Saito, Kaori Sano, Tadaki Suzuki, Akira Ainai, Yuki Taga, Tomonori Ueno, Koshiro Tabata, Kumpei Saito, Yuji Wada, Yuki Ohara, Haruko Takeyama, Takato Odagiri, Tsutomu Kageyama, Kiyoko Ogawa-Goto, Pretty Multihartina, Vivi Setiawaty, Krisna Nur Andriana Pangesti, Hideki Hasegawa

**Affiliations:** 1 Department of Pathology, National Institute of Infectious Diseases, Shinjuku, Tokyo, Japan; 2 Influenza Virus Research Center, National Institute of Infectious Diseases, Musashimurayama, Tokyo, Japan; 3 Division of Infectious Diseases Pathology, Department of Global Infectious Diseases, Tohoku Graduate School of Medicine, Sendai, Miyagi, Japan; 4 Nippi Research Institute of Biomatrix, Toride, Ibaraki, Japan; 5 Department of Life Science and Medical Bioscience, Waseda University, Shinjuku, Tokyo, Japan; 6 National Institute of Health Research and Development, Ministry of Health RI, Jakarta, Indonesia; St. Jude Children's Research Hospital, UNITED STATES

## Abstract

Mucosal immunoglobulins comprise mainly secretory IgA antibodies (SIgAs), which are the major contributor to pathogen-specific immune responses in mucosal tissues. These SIgAs are highly heterogeneous in terms of their quaternary structure. A recent report shows that the polymerization status of SIgA defines their functionality in the human upper respiratory mucosa. Higher order polymerization of SIgA (i.e., tetramers) leads to a marked increase in neutralizing activity against influenza viruses. However, the precise molecular mechanisms underlying the effects of SIgA polymerization remain elusive. Here, we developed a method for generating recombinant tetrameric monoclonal SIgAs. We then compared the anti-viral activities of these tetrameric SIgAs, which possessed variable regions identical to that of a broadly neutralizing anti-influenza antibody F045-092 against influenza A viruses, with that of monomeric IgG or IgA. The tetrameric SIgA showed anti-viral inhibitory activity superior to that of other forms only when the antibody exhibits low-affinity binding to the target. By contrast, SIgA tetramerization did not substantially modify anti-viral activity against targets with high-affinity binding. Taken together, the data suggest that tetramerization of SIgA improved target breadth, but not peak potency of antiviral functions of the broadly neutralizing anti-influenza antibody. This phenomenon presumably represents one of the mechanisms by which SIgAs present in human respiratory mucosa prevent infection by antigen-drifted influenza viruses. Understanding the mechanisms involved in cross neutralization of viruses by SIgAs might facilitate the development of vaccine strategies against viral infection of mucosal tissues.

## Author summary

SIgAs exist as mainly dimers and tetramers and play critical roles in mucosal immune responses against influenza. Detailed characterization of these anti-viral SIgA is important for better understanding of the mechanisms underlying anti-viral immunity. Here, we describe a means of generating a recombinant tetrameric monoclonal SIgA to enable exhaustive characterization of tetrameric SIgAs. The tetrameric monoclonal SIgA possessing variable regions of anti-influenza viruses broadly neutralizing antibody show that tetramerization of SIgA improves target breadth, but not the peak potency, of their anti-viral functions. These results broaden our knowledge about the fundamental role of SIgA tetramerization in anti-viral humoral response at the human respiratory mucosa.

## Introduction

Secretory IgA antibodies (SIgAs) play an important role as a first line of defense by inactivating pathogens on mucosal surfaces; this is especially true in the case of viruses such as influenza [1, 2]. Recently, extensive efforts were made to develop novel vaccines that induce immunity via the mucosal route. SIgA is the major contributor to humoral mucosal immunity and is a key molecule that underpins the action of mucosal vaccines [3, 4]. Therefore, understanding how SIgA works is important if we are to accelerate development of mucosal vaccines.

IgA is the major immunoglobulin isotype in humans; indeed, its production exceeds that of all other immunoglobulin classes combined [2]. In addition, IgA displays a number of features that make it unique among the immunoglobulin classes; the most characteristic of these is its quaternary structure [5]. Most of the IgA in human serum is monomeric (comprising two α heavy (H) and two light (L) chains). IgA present in external secretions is highly heterogeneous, although the majority is present in the form of polymers in which the heavy chains are covalently linked by a J chain. Moreover, these polymeric IgA antibodies are associated with the extracellular portion of the polymeric immunoglobulin receptor (pIgR), called the secretory component (SC), resulting in SIgA [5]. SIgA is composed primarily of dimers, although some larger polymeric forms, particularly tetramers, are present at low levels [5–10]. These tetrameric SIgA antibodies display greater neutralizing activity against influenza A viruses in the nasal mucosa than monomers or dimers [8, 9]. However, the molecular mechanisms that underlie these characteristics of tetrameric SIgA remain largely unknown. Therefore, to elucidate these molecular mechanisms and evaluate the impact of SIgA polymerization on protection against viral infections, it is essential to obtain IgA antibodies as monomers, dimers, and tetramers that display identical variable regions; only in this way can we make a fairly accurate comparison of their functions. Although several *in vitro* methods of generating recombinant polymeric IgA have been reported, they focus mainly on producing dimeric IgA [11–13] rather than tetramers. No one has yet developed a method of generating trimeric or tetrameric IgA molecules.

Here, we developed a method of generating recombinant monoclonal human tetrameric SIgAs by co-expressing human αH, L, and J chains plus the SC in mammalian cells. This simple method enabled us to examine the effects of SIgA polymerization on its anti-viral activity against influenza A viruses. We compared the reactivity and functionality of generated broadly neutralizing antibodies (bnAb) comprising monomeric IgA, dimeric SIgA, or tetrameric SIgA and found that SIgA polymerization led to a marked increase in activity against viruses to which the antibodies bind with low-affinity, but not with high-affinity at monomeric state. Taken together, the results suggest that SIgA polymerization improves target breadth, but not the peak potency of anti-viral functions of bnAbs against influenza A viruses.

## Results

### Co-expression of the SC along with human αH, L, and J chains in mammalian cells enables generation of recombinant tetrameric monoclonal SIgAs *in vitro*

**Fig 1:**
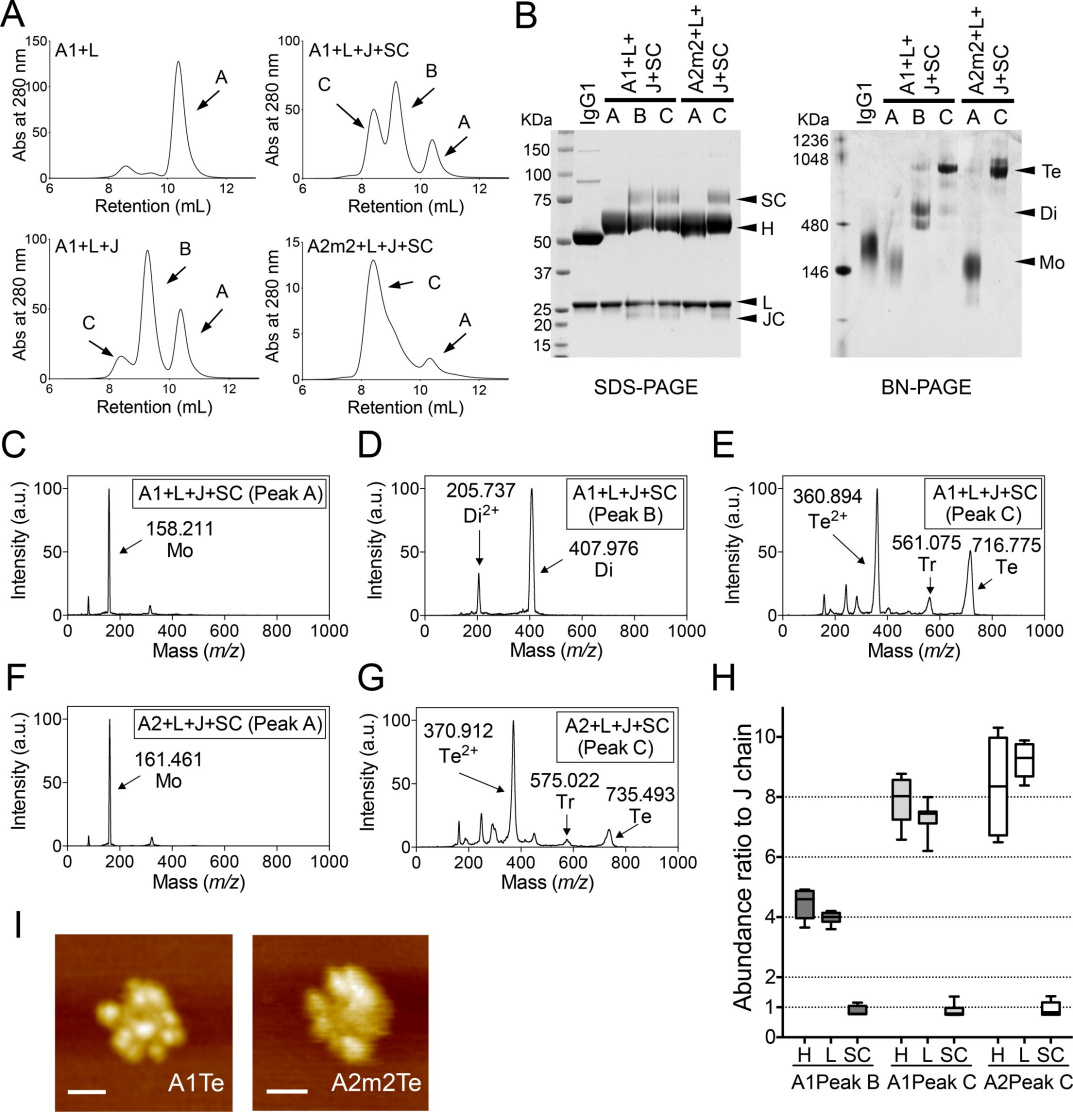
Production of recombinant tetrameric monoclonal SIgAs. (A) Recombinant monoclonal IgA antibodies purified from the culture supernatant of cells co-transfected with A1+L (left upper), A1+L+J (left lower), A1+L+J+SC (right upper), or A2m2+L+J+SC (right lower), were subjected to size exclusion chromatography (SEC) analysis. A chromatogram showing absorbance at 280 nm revealed three major peaks: peak A (retention volume around 10.4 ml), peak B (retention volume around 9.3 ml), and peak C (retention volume around 8.4 ml). Data are representative of three independent experiments. (B) SDS-PAGE and BN-PAGE analysis of IgG and IgA1/IgA2m2 in each peak fraction (peak A, B, and C) purified from cells co-expressing SC (A1+L+J+SC or A2m2+L+J+SC). (C, D, E) High-mass MALDI-TOF MS analysis of the each peak fraction containing recombinant IgA1 purified from the culture supernatant of cells transfected with A1, L, J, and SC. (C) One main peak (arrow) corresponding to monomer (Mo) was detected in the peak A fraction. (D) Two main peaks (arrows) corresponding to a dimer (Di) and a di-cation dimer (Di^2+^) were detected in the peak B fraction. (E) Three main peaks (arrows) corresponding to a tetramer (Te), trimer (Tr), and di-cation tetramer (Te^2+^) were detected in the peak C fraction. (F, G) High-mass MALDI-TOF MS analysis of the each peak fraction of recombinant IgA2m2 purified from the culture supernatant from cells transfected with A2m2, L, J, and SC. (F) One main peak (arrow) corresponding to a monomer (Mo) was detected in the peak A fraction. (G) Three main peaks (arrows) corresponding to a tetramer (Te), a trimer (Tr), and a di-cation tetramer (Te^2+^) were detected in the peak C fraction. (H) Quantification of the amount of each subunit within the peak B or C fraction of recombinant SIgA1 or SIgA2m2 antibodies purified from the culture supernatant of cells transfected with A1/L/J,/SC or A2m2/L/J/SC using LC-MS with stable isotope-labeled standard peptides. The abundance of each subunit to that of J chain is expressed as a ratio. Data are expressed as box-and-whisker plot with minimum, maximum, median, upper and lower quartiles (n = 6–7). (I) HS-AFM image of peak C derived from a recombinant SIgA1 (A1Te) or SIgA2m2 (A2m2Te) antibody purified from the culture supernatant of cells transfected with A1/L/J/SC or A2m2/L/J/SC. Scale bar, 20 nm.

Previous studies report that co-expression of αH, L, and J chains in mammalian cells results in formation of dimeric IgA [11–14]. However, production of polymeric IgA antibodies larger than dimers is restricted [14]. Indeed, even when researchers succeeded in generating some polymeric antibodies larger than dimers, they were not well characterized [13]. In general, trimeric or tetrameric forms of IgA are present in external sections as secretory forms containing a SC. Therefore, to generate polymeric IgA antibodies in secretory form, the SC was co-expressed in mammalian cells along with human α1 heavy (A1), L, and J chains. Next, IgA antibodies purified from the cell culture supernatant were separated by size exclusion chromatography (SEC). SEC analysis revealed that co-expression of A1, L, and J chains produced IgA antibodies with three different quaternary structures, corresponding to peak A (retention volume around 10.4 mL), peak B (retention volume around 9.3 mL), and peak C (retention volume around 8.4 mL). Peak C (with the lowest retention volume corresponding to the largest molecule size) was increased to a significantly greater extent upon co-expression of the SC (Fig 1A). Furthermore, co-expression of the SC along with a α2 heavy (A2m2) chain instead of A1 (another subclass of human IgA) led to a marked increase in production of molecules corresponding to peak C and a reduction in production of those corresponding to peak B (Fig 1A). To characterize these polymeric forms of recombinant IgA1 and IgA2m2, we analyzed each peak fraction containing IgA antibodies co-expressed with the SC by sodium dodecyl sulfate polyacrylamide gel electrophoresis (SDS-PAGE), and Blue Native polyacrylamide gel electrophoresis (BN-PAGE). SDS-PAGE revealed that the peak fractions corresponding to peaks B and C comprised A1/A2m2, L, and, J chains plus the SC, suggesting that these antibodies were secretory forms. By contrast, the fraction corresponding to peak A lacked the J chain and the SC (Fig 1B, left panel). BN-PAGE analysis showed that IgA1 in peak B had a molecular weight of around 500 KDa, whereas peak C mainly contained proteins with molecular weight >720 KDa (Fig 1B, right panel). The band observed in peak B was not detected in peak C from IgA2m2, although the band corresponding to the protein with a molecular weight >720 KDa was detected (Fig 1B, right panel). To determine the molecular size of the recombinant IgA antibodies more accurately, each peak fraction of IgA1 or IgA2m2 (derived from A1/L/J/SC- or A2m2/L/J/SC-expressing cells, respectively) was examined by high-mass MALDI-TOF MS. IgA1 samples yielded peaks corresponding to four quaternary structures: a monomer (Mo, MH+ = 158.211 ± 0.163 kDa) in peak A (Fig 1C), a dimer (Di, MH+ = 407.976 ± 0.603 kDa) in peak B (Fig 1D), a trimer (Tr, MH+ = 561.075 ± 0.678 kDa) in peak C, and a tetramer (Te, MH+ = 716.775 ± 0.879 kDa) in peak C (Fig 1E). By contrast, IgA2m2 yielded peaks corresponding to three quaternary structures: a monomer (Mo, MH+ = 161.461 ± 0.190 kDa) in peak A (Fig 1F), a trimer (Tr, MH+ = 575.022 ± 0.633 kDa) in peak C, and a tetramer (Te, MH+ = 735.493 ± 0.941 kDa) in peak C (Fig 1G). Although high-mass MALDI-TOF MS analysis is only a qualitative, and not a quantitative method, the peaks corresponding to the trimer in peak C were much smaller than those corresponding to the tetramer in peak C; this was true for both IgA1 and IgA2m2, implying that the major quaternary structure in peak C was tetramer (for both IgA1 and IgA2m2). To rule out the possibility that the peak C fraction comprised aggregates of dimeric SIgA, we determined the ratios of each SIgA1 or SIgA2m2 subunit in the peak B or C fraction. We performed LC-MS analysis of marker peptides selected for respective IgA subunits after addition of corresponding stable isotope-labeled internal standard peptides followed by trypsin digestion. The ratio of H:L:J:SC of the peak C derived from both of IgA1 and IgA2m2 was approximately 8:8:1:1, whereas that of peak B derived from IgA1 was approximately 4:4:1:1 (Fig 1H). This was consistent with a previous report of human tetrameric IgA [6], and confirmed that majority of polymeric IgA molecules in the peak C fraction were tetrameric, and not dimeric (ratio, 4:4:1:1) or trimeric (assumed ratio, 6:6:1:1). Therefore, the peak A, B, or C fractions generated by co-expression of SC and A1/A2m2, L, and J chains were labeled monomeric, dimeric, or tetrameric, respectively. For further confirmation of the structures of this recombinant tetrameric SIgA antibody in peak C, we visualized the quaternary molecular architecture of the SIgA1 (A1Te) or SIgA2m2 (A2m2Te) molecules in the peak C fraction using high-speed atomic force microscopy (HS-AFM). HS-AFM revealed that peak C contained molecules with eight radial arms (A1Te) or more than six arms (A2m2Te), a similar quaternary structure to that of tetrameric SIgA derived from human nasal mucosa, but not similar to trimeric IgA; this also suggests most of the molecules in this fraction were tetrameric (Fig 1I). Taken together, the data show that co-expression of SC along with IgA1/IgA2m2 heavy, light, and J chains in mammalian cells increases production of recombinant tetrameric monoclonal SIgAs, which possess a characteristic quaternary structure corresponding to tetrameric SIgAs found in human external secretions.

### SIgA polymerization increases the binding activity of bnAb against influenza A viruses

In a previous study, we showed that trimeric/tetrameric SIgAs in the human nasal mucosa display greater neutralizing activity against influenza A viruses than monomeric immunoglobulins [8, 9]. To reveal the mechanism underlying this polymerization-mediated antibody activity enhancement, recombinant monomeric, dimeric or tetrameric monoclonal SIgAs possessing variable regions of antibody clone F045-092, a bnAb against influenza A viruses [15, 16], was prepared. It was reported by Ohshima et al. that clone F045-092 IgG possesses binding activity against all H3N2 viruses isolated during 1968 to 2004, as well as some H1N1, H2N2, and H5N1 viruses [16]. The antibody recognizes the receptor-binding domain on the hemagglutinin (HA) protein of influenza A viruses and shows hemagglutination inhibition (HI) activity and neutralization (NT) activity [15, 16]. Antibodies with HI activity block the interaction between the receptor-binding domain located on the HA head and its sialic-acid receptor [17]. In addition, HI activity is the main component of anti-influenza virus immunity *in vivo*, and correlates with the level of protection against influenza in humans.

**Fig 2:**
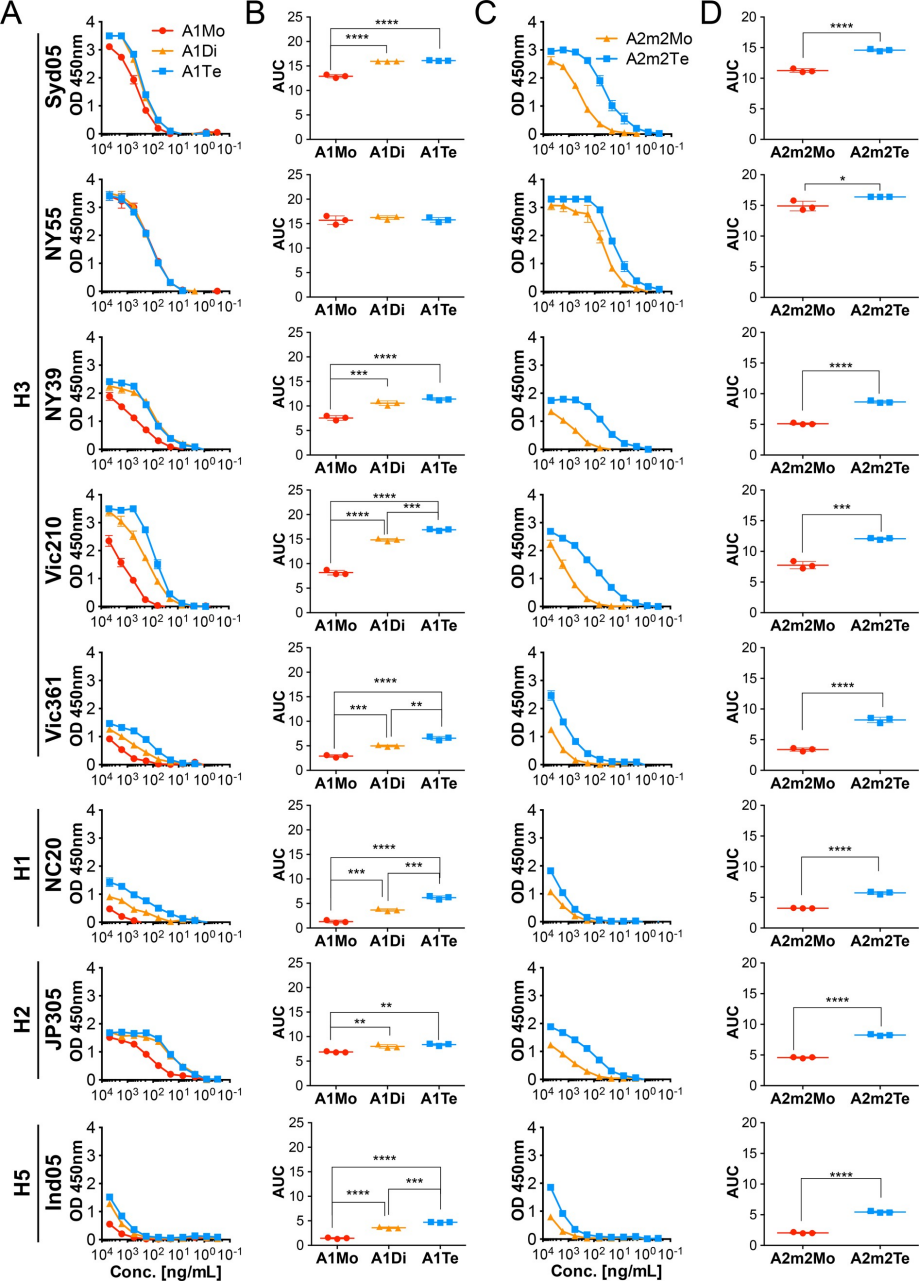
Reactivity of F045-092 bnAb against influenza A viruses on IgA backbones with or without polymerization. (A) Reactivity of monomeric (red, A1Mo), dimeric (orange, A1Di), and tetrameric (blue, A1Te) F045-092 IgA1 antibodies against recombinant HA proteins from A/Sydney/05/97 (H3N2; Syd05), A/New York/55/2004 (H3N2; NY55), A/New York/39/2012 (H3N2; NY39), A/Victoria/210/2009 (H3N2; Vic210), A/Victoria/361/2011 (H3N2; Vic361), A/New Caledonia/20/99 (H1N1; NC20), A/Japan/305/2957 (H2N2, JP305), and A/Indonesia/5/2005 (H5N1, Ind05) viruses. Data are expressed as the mean ± SD of three technical replicates. (B) Area under the reactivity curve (AUC) for each IgA1 antibody (A1Mo, A1Di, and A1Te) tested against each HA. The AUC was calculated from the plots in (A). Data are expressed as the mean ± SD of three technical replicates. (C) Reactivity of monomeric (red, A2m2Mo) and tetrameric (blue, A2m2Te) F045-092 IgA2m2 antibodies against recombinant HA proteins from Syd05, NY55, NY39, Vic210, Vic361, NC20, JP305, and Ind05 viruses. Data are expressed as the mean ± SD of three technical replicates. (D) AUC for the IgA2m2 antibody (A2m2Mo, and A2m2Te) tested against each HA. The AUC was calculated from the plots in (C). Data are expressed as the mean ± SD of three technical replicates. *p<0.05, **p<0.01, ***p<0.001, and ****p<0.0001 (unpaired Student t-test or one-way ANOVA followed by Tukey’s multiple comparison test).

At first, to determine whether polymerization of monoclonal SIgAs influences their reactivity with HA proteins of influenza A viruses, we examined the reactivity of monomeric IgA1 (A1Mo), dimeric SIgA1 (A1Di), tetrameric SIgA1 (A1Te), monomeric IgA2m2 (A2m2Mo), and tetrameric SIgA2m2 (A2m2Te) harboring identical variable regions derived from F045-092 against recombinant HA proteins from A/Sydney/05/97 (H3N2; Syd05), A/New York/55/2004 (H3N2; NY55), A/New York/39/2012 (H3N2; NY39), A/Victoria/210/2009 (H3N2; Vic210), A/Victoria/361/2011 (H3N2; Vic361), A/New Caledonia/20/99 (H1N1; NC20), A/Japan/305/1957 (H2N2; JP305), and A/Indonesia/05/2005 (H5N1; Ind05) by Enzyme-linked immunosorbent assay (ELISA). The reactivity of IgA1 antibodies to HA proteins increased significantly in line with molecular size (the exception was NY55 HA) (Fig 2A and 2B). In particular, the reactivity of tetrameric SIgA1 against Vic210 HA, Vic361 HA, NC20 HA, and Ind05 HA was significantly higher than that of dimeric SIgA1. By contrast, the three forms of IgA1 antibodies (Mo, Di, and Te) showed no difference in reactivity to NY55 HA; all three showed good potency, indicating that the effects of SIgA1 polymerization might be limited by the binding mode between the epitope and paratope. Meanwhile, A2m2Te showed markedly higher reactivity against HA proteins of all viruses than A2m2Mo (Fig 2C and 2D). Increase in reactivity of F045-092 IgA1 and IgA2m2 by polymerization could also be observed against whole virions of NC20 virus (S1 Fig). These results suggest that tetramerization boosts the avidity of the F045-092 SIgA antibody to HA on the virions.

**Fig 3:**
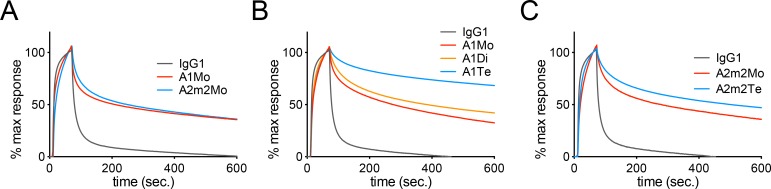
Binding dynamics analysis of IgG and IgA antibodies by surface plasmon resonance (SPR) analysis. (A) F045-092 IgG1 (black), monomeric IgA1 (red, A1Mo), and monomeric IgA2m2 (blue, A2m2Mo) antibodies were subjected to SPR analysis of their binding dynamics to the recombinant trimeric HA proteins from JP305 virus (H2N2). (B) Binding dynamics of F045-092 IgG1 (black), monomeric IgA1 (red, A1Mo), dimeric SIgA1 (orange, A1Di), and tetrameric SIgA1 (blue, A1Te) antibodies to the recombinant trimeric HA proteins from JP305 virus (H2N2) were examined. (C) Binding dynamics of F045-092 IgG1 (black), monomeric IgA2m2 (red, A2m2Mo), and tetrameric SIgA2m2 (blue, A2m2Te) antibodies to the recombinant trimeric HA proteins from JP305 virus (H2N2) were examined. Sensorgrams were x and y-axis adjusted (x = 0, y = 0: baseline, y = 100:binding) to allow comparisons between different antibody forms in terms of the dissociation rate of IgA from HA.

In order to fairly compare binding characteristics of F045-092 IgG1, A1, and A2m2 against a recombinant HA protein, we examined the binding dynamics of these antibodies to JP305 HA by surface plasmon resonance (SPR). A1Mo and A2m2Mo dissociated less well from JP305 HA than IgG1 (Fig 3A). In addition, A1Te and A2m2Te dissociation rates were evidently lower than those of their smaller molecular sizes, A1Mo, A1Di, or A2m2Mo, respectively (Fig 3B and 3C). Thus, although isotype conversion to IgA backbone from IgG backbone influenced the antibody reactivity, IgA tetramerization boosted the antibody avidity of IgA molecules, which is likely to be through different mechanisms from the effect by the isotype conversion from IgG to IgA backbone.

### Effects of SIgA polymerization on the inhibitory activity of bnAb against influenza A viruses

Polymerization of the F045-092 antibody using an IgA backbone increased its reactivity with several HA proteins. However, the biological activity of antibodies specific for influenza viruses, which is designated as the “functionality” of an antibody here, does not necessarily correspond to the binding activity observed in an ELISA, which is designated as the “reactivity” of an antibody here. The HI and NT assays are the most widely accepted assays for measuring the functional activity of antibodies against influenza viruses [18]. Although both assays measure the functionality of inhibitory antibodies, they measure immune responses in different ways. The NT assay measures the ability of antibodies to inhibit virus infection of mammalian host cells. By contrast, HI activity correlates only with the ability of antibodies to inhibit virus attachment to host cells via sialic-acid receptors; thus, the readout from the HI assay emphasizes steps that occur very early during infection [17]. Therefore, the two activities do not necessarily correlate, and the difference depends on how the antibody interacts with its target antigen.

**Fig 4:**
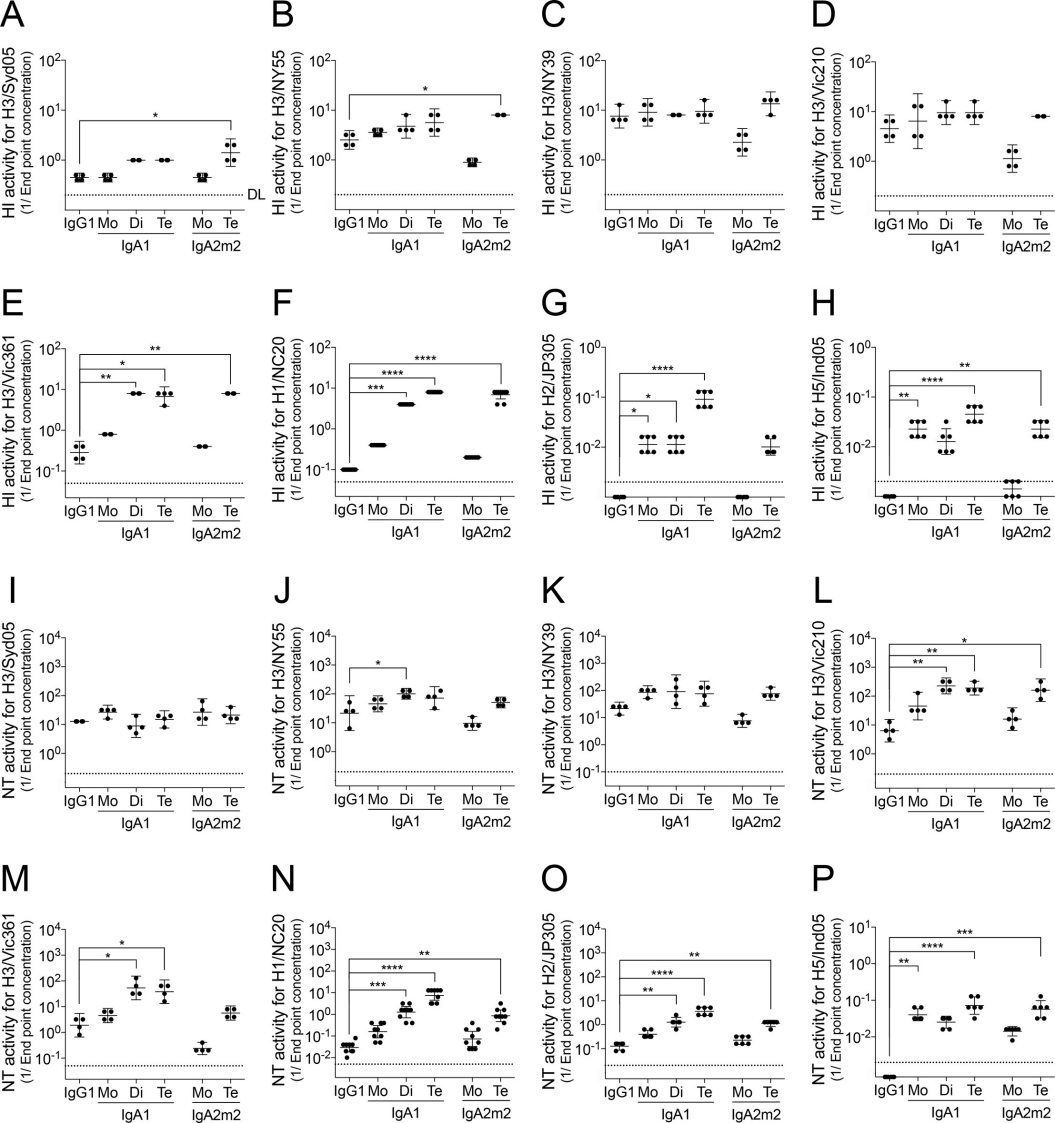
Hemagglutinin inhibition and neutralizing activities of F045-092 on IgG and IgA, with or without polymerization. (A–H) HI activity of F045-092 IgG1, monomeric (Mo) IgA1, dimeric (Di) SIgA1, tetrameric (Te) SIgA1, monomeric (Mo) IgA2m2, tetrameric (Te) SIgA2m2 against Syd05 (A), NY55 (B), NY39 (C), Vic210 (D), Vic361 (E), NC20 (F), JP305 (G), or Ind05 (H) viruses. HI activity is shown on scatter plots as the geometric mean (with 95% confidence intervals) of the reciprocal of the lowest concentration (μg/ml) of antibody that inhibited hemagglutination of the virus (n = 4 for H3 viruses, n = 6 for JP305 and Ind05 viruses, and n = 9 for NC20 virus). (I–P) NT activity of F045-092 IgG1, monomeric (Mo) IgA1, dimeric (Di) SIgA1, tetrameric (Te) SIgA1, monomeric (Mo) IgA2m2, and tetrameric (Te) SIgA2m2 against Syd05 (I), NY55 (J), NY39 (K), Vic210 (L), Vic361 (M), NC20 (N), JP305 (O), or Ind05 (P) viruses. NT activity is expressed on scatter plots as the geometric mean (with 95% confidence intervals) of the reciprocal of the lowest concentration (μg/ml) of antibody that neutralized the virus (n = 4 for H3 viruses, n = 6 for JP305 and Ind05 viruses, and n = 9 for NC20 virus). The dotted line in the graph represents the detection limit (DL) of each experiment. Half of the detection limit value was applied to samples with titers below the detection limit for statistical analyses. *p<0.05, **p<0.01, ***p<0.001, and ****p<0.0001 (compared with IgG; Kruskal-Wallis test followed by Dunn’s multiple comparison test).

The effect of SIgA polymerization on HI activity of F045-092 differed by virus strain and IgA antibody subclass. Here, we found that the HI activities of A2m2Te against Syd05 or NY55 viruses were significantly higher than that of the F045-092 antibody with an IgG1 backbone (Fig 4A and 4B). In addition, the HI activity of all of polymerized SIgAs (A1Di, A1Te, and A2m2Te) against Vic361 or NC20 viruses was markedly higher than that of IgG1 (Fig 4E and 4F). By contrast, the HI activity of SIgAs against NY39 or Vic210 viruses was similar to that of IgG1 (Fig 4C and 4D).

The degree of NT activity enhancement by SIgA polymerization also differed by the virus strain used and the IgA antibody subclass. The NT activity of the polymerized SIgA against Syd05 or NY39 viruses was no different from that of IgG1 (Fig 4I and 4K), but NT activity against Vic210 or NC20 viruses was significantly higher than that of IgG1 (Fig 4L and 4N). However, only the IgA1 polymer (A1Di or A1Te) showed higher NT activity against NY55 or Vic361 viruses than IgG1 (Fig 4J and 4M).

Enhancement in functionality of F045-092 by SIgA polymerization could also be seen against the JP305 and Ind05 viruses, each of the H2N2 and H5N1 subtype, respectively (Fig 4G, 4H, 4O and 4P). Of note, for JP305 and Ind05 virus strains, against which F045-092 IgG1 did not possess HI activity, significant increases in HI activity could be observed by just converting F045-092 from IgG1 to A1Mo form (Fig 4G and 4H). A significant increase could also be observed in NT activity against Ind05 virus by the same conversion (Fig 4P). This may be, in part, due to the difference in F045-092 avidity to HA between IgG1, and IgA monomers including A1Mo and A2m2Mo (Fig 3A). Furthermore, virus neutralization experiments in the mouse respiratory tract indicated that there is a slight possibility that polymeric SIgA have additive effects on protection from influenza virus infection *in vivo* even in the absence of increased NT activity *in vitro* (S2 Fig). Taken together, the results suggest that although there are some differences in the levels of functional enhancement in the HI or NT assays, polymerization of SIgA led to a significant increase in functionality against some viruses.

### SIgA polymerization leads to improvement in target breadth of bnAb against influenza A viruses

**Fig 5:**
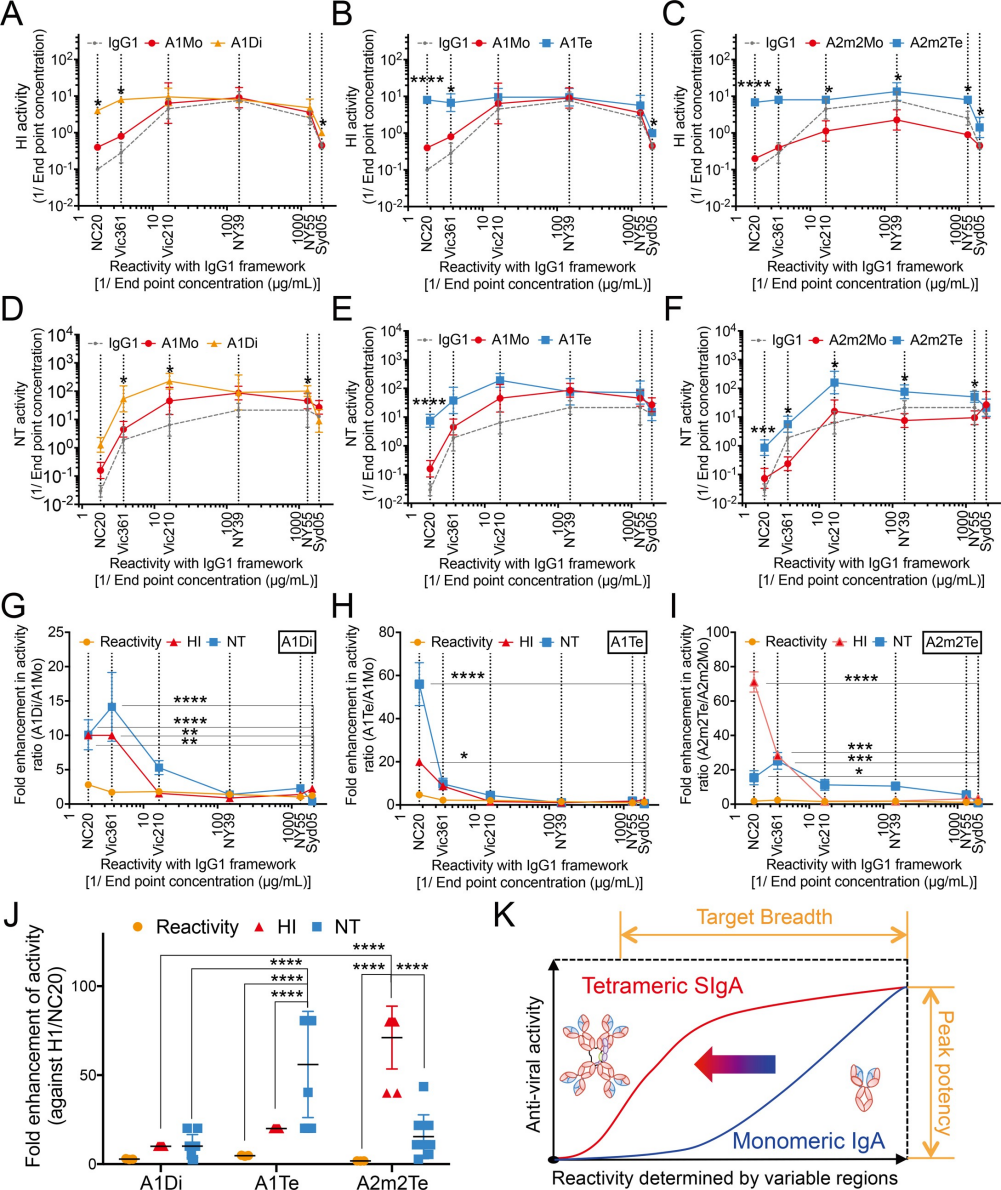
Relationship between increased functionality and reactivity of polymerized SIgA. (A–F) Line graphs depict the relationship between hemagglutinin inhibition (A–C) or neutralizing (D–F) activity of IgG1 (gray dotted line in A–F), monomeric IgA1 (A1Mo; red line in A, B, D and E), dimeric SIgA1 (A1Di; orange line in A and D), tetrameric SIgA1 (A1Te; blue line in B and E), monomeric IgA2m2 (A2m2Mo; red line in C and F), and tetrameric SIgA2m2 (A2m2Te; blue line in C and F) and reactivity against each virus HA. The HI and NT activity against Syd05, NY55, NY39, Vic210, Vic361, and NC20 viruses, against which F045-092 possessed both HI and NT activity at IgG1 state as shown in Fig 4, are integrated into the graphs. The Y-axis shows HI or NT activity and the X-axis shows the reactivity value, expressed as the geometric mean of the reciprocal of the lowest concentration (μg/ml) of F045-092 IgG1 that binds to each viral HA. Vertical dotted lines denote the reactivity value against the virus indicated below the X-axis. The each symbol on the vertical dotted line represents the geometric mean (with 95% confidence intervals) of the HI or NT activity against the virus indicated below the X-axis. *p<0.05, **p<0.01, ***p<0.001, and ****p<0.0001 (compared with monomeric IgA; Holm-Sidak t-test). (G–I) The line graphs depict the relationship between reactivity (determined by the IgG1 backbone) against each viral HA protein and the ratio of reactivity (orange line), HI activity (red line), or NT (blue line) activity of A1Di (G), A1Te (H), or A2m2Te (I) to that of monomeric IgA (A1Di/A1Mo, A1Te/A1Mo, or A2m2Te/A2m2Mo). The X-axis shows the reactivity value, expressed as the geometric mean of the reciprocal of the lowest concentration (μg/ml) of F045-092 IgG1 that bound to each viral HA protein. Vertical dotted lines show reactivity against the virus indicated below the X-axis. Each symbol on the vertical dotted line represents the mean ± SEM. *p<0.05, **p<0.01, ***p<0.001, and ****p<0.0001 (functional activity of antibodies against each virus compared with that against the Syd05 virus; two-way ANOVA followed by Dunnett's multiple comparison test). (J) Increase of reactivity, HI, and NT activity against NC20 virus induced by SIgA1 dimerization, SIgA1 tetramerization, and SIgA2m2 tetramerization. Data are expressed as the mean ± SD. ****p<0.0001 (two-way ANOVA followed by Tukey's multiple comparisons test). (K) A hypothesis-explanatory drawing illustrating the effect of SIgA polymerization on antibody function. Polymerization of both IgA subclasses increased anti-viral activity against influenza A viruses. Increased the anti-viral activity emerged strikingly to the viruses with low-affinity binding resulting in increased breadth, but not peak potency, of the anti-viral activity of antibody.

The effects of SIgA polymerization on functionality against influenza A viruses were more complex; Fig 4 shows that some viruses were more susceptible to SIgA polymers than others. The degree of enhancement may be defined by the antibody clone’s inherent binding affinity to various HA molecules. To clarify the relationship between reactivity and altered anti-viral activity shown in Fig 4 induced by SIgA polymerization, we plotted the HI and NT activities of IgG1, IgA monomer, and IgA polymers against six viruses ordered based on the reactivity to F045-092 with an IgG1 backbone, which is defined here as the “original” reactivity. Syd05, NY55, NY39, Vic210, Vic361, and NC20 viruses, against which F045-092 possessed both HI and NT activity at the IgG1 form, were included in the analyses. As a result, we found that the polymerized antibodies showed increased anti-viral activity (i.e., HI and NT activity) against the viruses to which they originally bound with relatively low-affinity (Fig 5A, 5B, 5D and 5E). By contrast, tetramerization of SIgA2m2 increased its anti-viral activity against almost all viruses when compared with the monomeric IgA2m2 antibody, although the monomeric IgA2m2 antibody showed lower activity than the IgG1 form; this suggests that the IgA2m2 backbone has some functional disadvantages compared to the IgG1 or IgA1 backbones, and that tetramerization of SIgA2m2 might overcome the disadvantages inherent in the IgA2m2 backbone (Fig 5C and 5F). To determine more accurately how SIgA polymerization affects reactivity and functionality of antibodies, we measured the increase in reactivity and anti-viral activity (HI or NT activities) and compared them with those of the monomeric forms. The data clearly show that SIgA1 dimerization increased anti-viral activity (HI and NT activities), and that tetramerization of SIgA1 and SIgA2m2 led to a significant increase in anti-viral activity against viruses with low original reactivity. Polymerization of SIgAs had a negligible effect on anti-viral activity against viruses with relatively high original reactivity such as Syd05, NY55, NY39, and Vic210 when compared with that against viruses with low original reactivity, such as Vic361 and NC20 (Fig 5G, 5H and 5I). By contrast, no significant difference in the effect of IgA polymerization on antibody reactivity could be observed among the six viruses (Fig 5G, 5H and 5I). In addition, the effects of SIgA1 or SIgA2m2 tetramerization on anti-viral activities against the NC20 virus, which exhibited the lowest original reactivity against, were significantly greater than the effects of SIgA1 dimerization (Fig 5J). Furthermore, SIgA1 tetramerization led to a higher-fold increase in NT activity than in HI activity against the NC20 virus, although SIgA1 dimerization led to a similar degree of increases in NT and HI activity (Fig 5J). On the other hand, SIgA2m2 tetramerization led to a higher-fold increase in HI activity rather than in NT activity against the NC20 virus. The differing effects of polymerization on SIgA1 and SIgA2m2 functionality suggest that each subclass of polymerized SIgA has a distinct mechanism of action when it comes to inhibiting influenza A virus infection.

To summarize, tetramerization of both IgA subclass increased anti-viral activities against influenza A viruses. These functional enhancements of antibodies appeared striking against viruses to which antibodies showed relatively low original reactivity, and no evident functional enhancements could be observed against viruses to which antibodies showed relatively high original reactivity. The number of antigen binding sites per antibody molecule did not simply correlate with the degree of increase in antibody reactivity nor functionality. Taken together, polymerization of a bnAb IgA antibody leads to an increase in its target breadth, but not in its peak potency of anti-viral functional activities (Fig 5K).

## Discussion

In the present study, we show that tetrameric monoclonal SIgA antibodies could be successfully generated by co-expression of antibody chains with the human SC in a mammalian cell line. Previous studies have shown that co-expression of αH, L, and J chains *in vitro* induces production of polymeric IgA, which comprises mainly dimeric IgA and some multimeric IgA antibodies [11–14]. However, these multimeric IgA antibodies were not characterized in detail, and differences in function between dimeric and multimeric IgA antibodies have remained obscure. The method described in the present study enables efficient production of recombinant tetrameric SIgA monoclonal antibodies, which enabled detailed examination of the molecular features and functions of SIgA antibodies of different valence.

The observation that the co-expression of A2, L, and J chains and the SC enhances the production of polymeric SIgA antibodies is inconsistent with previous reports [11, 12] showing that stable transfection of a mouse myeloma cell line (Sp2/0) expressing human SC and mouse-human chimeric dimeric IgA1 produced dimeric but not tetrameric SIgA [12]. In addition, CHO cells constitutively expressing human A2, L, and J chains and the SC also produced dimeric SIgA but no tetrameric SIgA [11]. However, these studies used stable cell lines constitutively expressing antibodies; these were obtained by a repeated cloning process in which cells expressing dimeric SIgA were selected. These methods may emphasize production of dimeric SIgA alone and create bias toward selecting cell clones that do not produce multimers larger than dimers. On the other hand, some IgA-producing hybridomas secrete trimeric and tetrameric IgA spontaneously in the absence of the J chain and SC [19, 20], indicating that αH chains have the intrinsic ability to form tetramers. However, plasma cells secreting polymeric IgA *in vivo* synthesize the J chain covalently linked to the αH chain [21], and IgA-producing cells in mucosal tissues express the J chain whereas IgA-positive cells in normal bone marrow do not [22]. These conflicting observations indicate that tetramerization of IgA may vary according to the intracellular environment. Here, we used a transient expression system based on Expi293F human cells derived from the 293 cell line. This system is specialized for recombinant protein expression and as such may not accurately and fairly reflect the molecular machinery involved in *in vivo* protein syntheses in specific types of cells, such as antibody-secreting cells, and may involve mechanisms distinct from the naive pathway used *in vivo* to generate tetrameric IgA antibodies. Although the molecular mechanism(s) responsible for the marked effects of SC co-expression on tetramer formation remain unclear (and appear to be artificial), we used sophisticated analytical chemistry techniques such as high-mass MALDI-TOF MS, HS-AFM, and LC-MS to show that tetrameric SIgAs obtained by this method have a molecular architecture similar to that of antibodies produced *in vivo*, and that they are fully functional in term of inactivating influenza A virus.

We previously reported that polymeric SIgAs larger than dimers play critical *in vivo* roles in protecting the human upper respiratory mucosa from virus attack, although the molecular mechanisms that underlie these particular characteristics of polymeric SIgA remain largely unknown [9]. The recombinant monoclonal IgA or SIgA (monomers, dimers, and tetramers) prepared herein served as fundamental tools for evaluating the impact of SIgA polymerization on anti-viral protection. Comparison of the reactivity and functionality of monomeric IgA, dimeric SIgA, and tetrameric SIgA F045-092 antibodies revealed that SIgA polymerization led to a marked increase in functionality against viruses to which the antibodies bound with relatively low affinity, but not with high affinity. This may suggest that in the case when the paratope and epitope of an antibody completely matches, the anti-viral activities against the virus will reach maximum level at monomeric state, leaving no room for further boosting of anti-viral activity by SIgA polymerization. Furthermore, against viruses with low affinity, the increase in functionality (e.g., HI and NT activity) due to tetramerization is much higher than the increase in reactivity, indicating that mechanisms other than increased avidity could be involved in functional enhancement. Therefore, polymerization of IgA may increase the breadth, but not the peak potency, of antibody function. In addition, recent reports present conflicting data on the effect of IgA polymerization; these reports focused on improving the functionality of bnAb against HIV1 by swapping the Fc region from IgG to IgA, and then polymerizing the IgA, though among these studies, the effect of IgA polymerization has remained controversial [23–29]. These reports used various viruses and bnAb antibody clones. Based on our observations that the degree of functional enhancement of IgA via polymerization might depend on the original affinity between the antibody and the antigen, we believe that the differences among data reported in the previous studies are due to differences in the viruses and clones used. Furthermore, the effect of dimerization of SIgA on antibody reactivity and functionality was markedly lower than that of tetramerization of SIgA (as evidenced by our observations), and previous reports did not examine the function of tetrameric forms.

Of note, in addition to the valence of IgA antibodies, the antibody subclasses and isotypes, which define the structure of the antibody backbone, may also influence the reactivity and functionality of antibodies [30, 31]. Human α1 and α2 H chains comprise one variable and three constant regions (CH1, CH2, and CH3), and harbor unique hinge regions between CH1 and CH2. The hinge region of IgA1 comprises 26 amino acids, whereas that of IgA2 comprises 13. The extended hinge region of IgA1 makes it highly susceptible to IgA1-specific proteases produced by several bacterial pathogens. However, this extended hinge provides greater segmental flexibility than that observed for IgA2. Thus, IgA1 may be better at binding antigens spaced at greater distances, which may enable recognition of pathogens (such as viral particles) possessing repeated antigenic structures spaced along their surfaces. Moreover, IgA1 accounts for most of the increase in anti-influenza virus IgA antibody titers observed in the human upper respiratory tract after influenza virus infection [32]. By contrast, antibodies in colostrum or saliva, which are specific for bacterial lipopolysaccharide and lipoteichoic acid, are predominantly IgA2, suggesting that the type of antigen may influence the subclass-specific IgA response [33]. The disadvantage of IgA2 antibodies in anti-viral activity could also be observed in the data presented in the current study. Whereas the HI and NT activities of monomeric IgA1 are higher than those of IgG1 (Fig 5B and 5E), the anti-viral activities of monomeric IgA2m2 are generally lower than those of IgG1 (Fig 5C and 5F). Since IgA2m2 could successfully boost these anti-viral activities by SIgA tetramerization, it could be assumed that SIgA tetramerization may be functioning as a way to overcome these functional disadvantages of monomeric SIgA2m2 antibodies. The fact that a significant increase in reactivity against H3 NY55 HA by SIgA tetramerization was only seen in IgA2m2 and not in IgA1 is also in accordance with such theories (Fig 2). In addition, we found that tetramerization of IgA1 and IgA2 increases different functions (HI activity and NT activity), which are distinct indicators of influenza virus infection (Figs 2, 5H and 5I). This suggests that each polymerized SIgA subclass has a distinct mechanism of action against influenza virus infections, and that tetrameric SIgA1 (which shows increased NT activity) might be more potent against influenza virus infections than tetrameric SIgA2. This may explain the molecular mechanisms underlying IgA subclass-specific immune responses to pathogens.

The observations made herein broaden our knowledge about the functions of tetrameric SIgA. Nevertheless, further work is needed if we are to obtain a more detailed picture of the functions of tetrameric SIgAs and the underlying molecular mechanisms. The method described herein may form the foundation for further studies designed to examine the impact of polymerizing antibody clones with different characteristics; this may enable us to generate bnAb against other virus infections including HIV and influenza viruses.

In conclusion, the comparison of reactivity and functionality among monomeric IgA, dimeric SIgA, and tetrameric SIgA monoclonal antibodies obtained by a novel method described herein revealed that SIgA tetramerization dramatically enhanced their functionality against viruses, to which the antibodies bind with relatively low affinity, but not with sufficient affinity, indicating SIgA tetramerization improves target breadth, but not peak potency of anti-viral functions of a bnAb against influenza A viruses. This phenomenon suggests the potential of SIgA antibodies to prevent infection of antigenically drifted influenza viruses at the human respiratory mucosa.

## Materials and methods

### Ethics statement

The study using embryonated chicken was carried out in compliance with the Standards Relating to the Care and Management of Laboratory Animals and Relief of Pain (the Ministry of Environment Notification No.88). The use of embryonated chicken eggs before hatching is not considered animal use. Embryonated eggs were purchased from Omiya Kakin Laboratory (Saitama, Saitama, Japan), inoculated with influenza viruses at day 8–11, incubated at 35°C for 2 days, and then incubated at 4°C overnight before allantoid fluid harvesting. For mice experiments, 7-week-old female BALB/c mice were purchased from Japan SLC (Hamamatsu, Shizuoka, Japan). Animal studies were performed in strict accordance with the Guidelines for Proper Conduct of Animal Experiments of the Scientific Council of Japan. All animal experiments were conducted in strict compliance with animal husbandry and welfare regulations in handled in biosafety level two animal facilities according to the guidelines of the Animal Care and Use Committee of the National Institute of Infectious Diseases, and were approved by this Committee (approval no. 118088). Mice were monitored daily for clinical signs of morbidity and mortality up to 21 days post infection. The human endpoint was used for mice that lost 30% or more of their initial body weight during the study. When the animals met the criteria, they were scored dead and euthanized under excess isoflurane anesthesia according to institutional guidelines.

### Viruses and cells

Madin-Darby canine kidney (MDCK, ATCC) cells were maintained in Dulbecco’s modified Eagle’s medium (DMEM, Gibco) containing 10% FBS (FBS, Gibco) and pen-strep mix (100 units/ml of penicillin and 100 μg/ml of streptomycin, Gibco) at 37°C/5% CO_2_ [34]. Expi293F (ThermoFisher Scientific) cells were maintained in Erlenmeyer flasks in Expi293 expression medium at 37°C/8% CO_2_. Influenza viruses were grown in 8–11-day-old embryonated chicken eggs. The virus strains used in this study were: A/Sydney/05/97 (H3N2; Syd05), A/New York/55/2004 (H3N2; NY55), A/New York/39/2012 (H3N2; NY39), A/Victoria/210/2009 (H3N2; Vic210), A/Victoria/361/2011 (H3N2; Vic361), A/New Caledonia/20/99 (H1N1; NC20), A/Japan/305/57 (H2N2; JP305), and A/Indonesia/05/2005 (H5N1; Ind05), and mouse adapted A/Guizou/54/1989 (H3N2; Gui54).

### Expression and purification of IgG and IgA

The plasmid harboring the α1H or α2H chain was derived from the γ1HC vector [35] by replacing the IgG1 constant domain with the human IgA1 or IgA2m2 constant domain, respectively. The DNA fragment encoding the human IgA1 constant domain was amplified by PCR using plasmid pFUSE-CHIg-hA1 (InvivoGen) as a template. The DNA fragment encoding the human IgA2m2 constant domain was codon-optimized (for humans) and synthesized using GeneArt Strings DNA Fragments (ThermoFisher Scientific). To establish the method used to produce tetrameric SIgA antibodies, the Fab regions derived from the B12 clone were used. The DNA fragment encoding the variable region of the H or L chain of the B12 clone was amplified by PCR using cDNA obtained from a single-sorted B cell from a healthy adult volunteer. The DNA fragment encoding the variable region of the H or L chain of F045-092 [16] was codon-optimized and synthesized using GeneArt Strings DNA Fragments (ThermoFisher Scientific). These synthesized DNA fragments were cloned into α1H, α2H, γ1HC, or λLC vectors. Human J chain was synthesized using GeneArt Strings DNA Fragments (ThermoFisher Scientific) and cloned into the pCXSN vector [36]. Human SC, consisting of the extracellular domain of the polymeric immunoglobulin receptor [37], the thrombin recognition site, and a hexa-histidine affinity tag at the C-terminus was synthesized and cloned into the pCXSN vector. To generate IgG, Expi293F cells grown in Expi293 expression medium were diluted to 2.5×10^6^ cells/ml and transfected with 100 μg of plasmid (35 μg of γ1HC and 65 μg of λLC) using 267 μl of ExpiFectamine293 Transfection reagent per 100 ml of final culture volume. To generate IgA, Expi293F cells were diluted to 2.5×10^6^ cells/ml and transfected with 40 μg of αH, 40 μg of λLC, 20 μg of J chain, and 20 μg of SC using 267 μl of ExpiFectamine293 Transfection reagent in 100 ml of final culture volume. At 16–18 hours post-transfection, 500 μl of ExpiFectamin293 Transfection Enhancer 1 and 5 ml of ExpiFectamin293 Transfection Enhancer 2 were added. Then, 5–7 days later, the cell culture supernatants were centrifuged at 2,000×g and filtered to remove cell debris. The supernatants were then purified using CaptureSelect IgG-Fc (Hu) (ThermoFisher Scientific) or CaptureSelect IgA (ThermoFisher Scientific), according to the manufacturer’s instructions. To prepare monomeric IgA, dimeric SIgA, and tetrameric SIgA, IgA samples purified using CaptureSelect were subjected to gel filtration chromatography on a Superose6 10/300 GL column (GE Healthcare) or Superose6 Increase 10/300 GL column (GE Healthcare). The peak fractions corresponding to each structure were collected and concentrated using Amicon Ultracell (Millipore) centrifugation units with a cut-off of 30 kDa and the buffers were changed to 20 mM phosphate buffer (PB) (pH 7.4) using a Zeba Spin Desalting Column (ThermoFisher Scientific). Purified antibodies were analyzed by Blue Native polyacrylamide gel electrophoresis (BN-PAGE) on NativePAGE 3–12% Bis-Tris gels (ThermoFisher Scientific) and by sodium dodecyl sulfate polyacrylamide gel electrophoresis (SDS-PAGE) on NuPAGE 4–12% Bis-Tris gels (ThermoFisher Scientific). NativeMark (ThermoFisher Scientific) or Precision Plus Protein All Blue standards (Bio-Rad Laboratories, Inc.) were used as a molecular weight markers for BN-PAGE or SDS-PAGE, respectively. The SDS-PAGE gels were stained with SimplyBlue SafeStain (ThermoFisher Scientific).

### HA proteins

The mammalian cell-derived HA proteins used in this study were: H1 NC20, H3 NY39, H3 Vic361, H3 Vic210, H3 NY55, H3Syd05, H3 BJ353 (from A/Beijing/353/1989 (H3N2) virus), H2 JP305, and H5 Ind05. These HA proteins comprise the extracellular domain of HA C-terminally fused to the thrombin site, the trimeric Foldon of T4 fibritin, and a hexa-histidine affinity tag [38] or a Strep-tag II and a hexa-histidine affinity tag. HA proteins were expressed using the Expi293 Expression System (ThermoFisher Scientific), according to the manufacturer’s instructions. Briefly, Expi293F cells grown in Expi293 expression medium in Erlenmeyer flasks were diluted to 2.5×10^6^ cells/ml and transfected with 100 μg of a HA-encoding plasmid using 267 μl of ExpiFectamine293 Transfection reagent per 100 ml of culture volume. At 16–18 hours post-transfection, 500 μl of ExpiFectamin293 Transfection Enhancer 1 and 5 ml of ExpiFectamin293 Transfection Enhancer 2 were added. At Day 5, the medium was clarified by centrifugation at 2,000×g, filtered, and purified on a HisTrap excel column (GE Healthcare). HA proteins with a Strep-tag II were purified with Strep-Tactin Superflow resin (iba), according to the manufacturer’s instructions, after purification on a HisTrap excel column. The purified HA proteins were concentrated using Amicon Ultracell (Millipore) centrifugation units with a cut-off of 30 kDa and the buffer was changed to phosphate buffered saline (PBS) (pH 7.4). The HA proteins were stored at -80°C until use.

### Quantification of IgA subunits

Stable isotope-labeled peptides (summarized in Table 1) were custom synthesized by Anygen. The internal standard peptides were mixed into 1–2 μg of each SIgA sample in 100 mM Tris-HCl/1 mM CaCl_2_ (pH 7.6). The samples were reduced with 5 mM dithiothreitol (Wako) at 56°C for 30 min and subsequently alkylated with 25 mM iodoacetamide (Wako) at room temperature for 30 min. The samples were then digested with sequencing-grade-modified trypsin (Promega; 1:20 enzyme/substrate ratio) at 37°C for 16 h. After addition of formic acid to a final concentration of 1% and filtration through a 0.45 μm filter, the peptide solutions were analyzed by electrospray LC-MS using an ultra-high resolution quadrupole time-of-flight mass spectrometer maXis II (Bruker Daltonics) coupled to a Shimadzu Prominence UFLC-XR system (Shimadzu). Peptide separation was performed using a BIOshell A160 Peptide C18 HPLC Column (5 μm particle size, L × I.D. 150 mm × 2.1 mm; Supelco) at a flow rate of 500 μl/min and a column temperature of 80°C with a binary gradient as follows: 98% solvent A (0.1% formic acid) for 2 min, linear gradient of 2–50% solvent B (100% acetonitrile) for 4 min, 90% solvent B for 2 min, and 98% solvent A for 2 min. The MS scan was performed over an *m/z* range of 50–2500 with a frequency of 2 Hz using otofControl version 4.0 (Bruker Daltonics). The absolute amounts of respective SIgA subunits were estimated using the peak area ratio of selected marker peptides to that of the corresponding internal standard peptides.

**Table 1:** Marker peptides used for quantitation of IgA subunits.

	Peptide	*m/z*	*z*
HC (IgA1)_1	NFPPSQDASGDLYTTSSQLTLPATQCLAGK	1056.8433 ± 0.02	3
HC (IgA1)_1-IS	NFPPSQDASGDLYTTSSQLTLPATQCLAG**K**	1059.5147 ± 0.02	3
HC (IgA1)_2	DASGVTFTWTPSSGK	770.8675 ± 0.02	2
HC (IgA1)_2-IS	DASGVTFTWTPSSG**K**	774.8746 ± 0.02	2
HC (IgA2)_1	NFPPSQDASGDLYTTSSQLTLPATQCPDGK	1066.1628 ± 0.02	3
HC (IgA2)_1-IS	NFPPSQDASGDLYTTSSQLTLPATQCPDG**K**	1068.8342 ± 0.02	3
HC (IgA2)_2	DASGATFTWTPSSGK	756.8519 ± 0.02	2
HC (IgA2)_2-IS	DASGATFTWTPSSG**K**	760.8590 ± 0.02	2
LC (λ)_1	YAASSYLSLTPEQWK	872.4330 ± 0.02	2
LC (λ)_1-IS	YAASSYLSLTPEQW**K**	876.4401 ± 0.02	2
LC (λ)_2	SYSCQVTHEGSTVEK	856.3832 ± 0.02	2
LC (λ)_2-IS	SYSCQVTHEGSTVE**K**	860.3903 ± 0.02	2
LC (κ)_1	DSTYSLSSTLTLSK	751.8829 ± 0.02	2
LC (κ)_1-IS	DSTYSLSSTLTLS**K**	755.8900 ± 0.02	2
LC (κ)_2	TVAAPSVFIFPPSDEQLK	973.5171 ± 0.02	2
LC (κ)_2-IS	TVAAPSVFIFPPSDEQL**K**	977.5242 ± 0.02	2
J_1	SSEDPNEDIVER	695.3101 ± 0.02	2
J_1-IS	SSEDPNEDIVE**R**	700.3142 ± 0.02	2
J_2	CYTAVVPLVYGGETK	828.9187 ± 0.02	2
J_2-IS	CYTAVVPLVYGGET**K**	832.9258 ± 0.02	2
SC_1	VYTVDLGR	461.7532 ± 0.02	2
SC_1-IS	VYTVDLG**R**	466.7573 ± 0.02	2
SC_2	GSVTFHCALGPEVANVAK	619.6490 ± 0.02	3
SC_2-IS	GSVTFHCALGPEVANVA**K**	622.3204 ± 0.02	3

**K**: ^13^C_6_^15^N_2_-lysine. **R**: ^13^C_6_^15^N_4_-arginine. C: carbamidomethylated cysteine.

### Size exclusion chromatography (SEC) analysis

Purified antibodies were filtered using Cosmo spin filter H (Nacalai Tesque, Inc.) to remove precipitates or debris before SEC analysis. Then, the antibodies were passed through an Agilent Bio SEC-5 500 Å (7.8×300 mm) column (Agilent Technologies) coupled to an Agilent 1260 Infinity Bio-inert HPLC system (Agilent Technologies). Analyses were performed using 1 μg or more of each antibody sample, with a flow rate of 1 ml/min. PBS (pH 7.4) was used as an eluent. Data were analyzed using OpenLAB CDS ChemStation Edition (Agilent Technologies).

### High-mass MALDI-TOF MS analysis

MALDI-TOF MS analysis was performed using the CovalX HM4 interaction module, with a standard nitrogen laser focusing on mass ranges from 0 to 1,500 kDa [8]. Aliquots (20 μl) of each protein sample were pipetted to prepare 2-fold dilutions, each with a final volume of 10 μl. Aliquots (1 μl) of each obtained dilution were mixed with 1 μl of a matrix comprising a re-crystallized sinapinic acid matrix (10 mg/ml) in acetonitrile/water (1:1 vol/vol) and 0.1% TFA (K200 MALDI Kit; CovalX). After mixing, 1 μl of each sample was spotted onto the MALDI plate (SCOUT 384; Bruker). After crystallization at room temperature, the plate was introduced into the MALDI mass spectrometer and analyzed immediately in high-mass MALDI mode. The analysis was repeated in triplicate. The following parameters were applied: mass spectrometer, linear and positive mode; ion source 1, 20 kV; ion source 2, 17 kV; lens, 12 kV; pulse ion extraction, 400 ns; HM4 gain voltage, 3.14 kV; and HM4 acceleration voltage, 20 kV. The instrument was externally calibrated using clusters of BSA and IgG. Three spots per sample were analyzed (300 laser shots per spot). The presented spectrum corresponds to the sum of 300 laser shots. The MS data were analyzed using CovalX Complex Tracker analysis software, version 2.0.

### High speed atomic force microscopy (HS-AFM)

The HS-AFM experiments were performed using a Nano Explorer High-Speed atomic force microscope (Research Institute of Biomolecule Metrology Co., Ltd.) with an Ultra-Short Cantilever (USC-F1.2-k0.15; NanoWorld). The tetrameric SIgA antibodies [2 μg/ml (2 μl in 10 mM PB (pH7.4))] were adsorbed onto a mica surface (Ted Pella, Inc.), incubated for 10 min, washed with 20 μl of double-distilled water (DDW), and then subjected to time-lapse imaging in DDW. Images containing 200×200 pixels were obtained at a scan rate of one frame per second (fps). Images were analyzed using SPIP software (Image Metrology A/S).

### ELISA

Half-area flat-bottomed microtiter plates (Costar) were coated overnight at 4°C with either recombinant HA proteins (H1 NC20, H2 JP305, H3 NY39, H3 Vic361, H3 Vic210, H3 NY55, H3 Syd05, or H5 Ind05) or whole virions (H1 NC20). Plates were coated with 50 ng/well for recombinant HA proteins except for H5 Ind05 and 250 ng/well for H5 Ind05 recombinant HA proteins and H1 NC20 whole virions. Plates were blocked for 1 h at 37°C with 1% BSA in PBS (pH 7.4) and serially diluted antibody samples were added to each well. Following incubation for 2 h at 37°C, wells were washed three times with PBS containing 0.05% Tween 20. After addition of diluted HRP-conjugated goat anti-human IgA antibody (Bethyl), HRP-conjugated mouse anti-human IgA2 antibody (abcam) or HRP-conjugated goat anti-human IgG-Fc fragment antibody (Bethyl) plates were incubated for 1 h at 37°C, washed three times, and incubated with One-Step Ultra TMB ELISA HRP substrate solution (Thermo Fisher Scientific). The reaction was stopped with H_2_SO_4_. Absorbance at 450 nm (reference: 655 nm) was measured in an iMark Microplate Reader (Bio-Rad). ELISA binding was expressed in terms of the area under the curve (AUC) as it better captures changes in both affinity and maximum binding of each antibody [39–41]. Curves and AUCs were constructed using GraphPad Prism software. The reactivity value was defined as the reciprocal of the lowest concentration (μg/ml) of antibody that bound to each viral HA protein. The titers of mice serum IgG specific for the HA proteins of H3 BJ353 virus, which was isolated in the same year as the challenge virus, A/Guizou/54/1989 (H3N2; Gui54) virus, were determined by ELISA. Half-area flat-bottomed microtiter plates (Costar) were coated (250 ng/well) overnight at 4°C with the HA proteins. Plates were blocked for 1 h at 37°C with 1% BSA in PBS (pH 7.4) and serially diluted serum samples were added to each well. Following incubation for 2 h at 37°C, wells were washed three times with PBS containing 0.05% Tween 20. After addition of diluted HRP-conjugated goat anti-mouse IgG-Fc fragment antibody (Bethyl) plates were incubated for 1 h at 37°C, washed three times, and incubated with One-Step Ultra TMB ELISA HRP substrate solution (Thermo Fisher Scientific). The reaction was stopped with H_2_SO_4_. Absorbance at 450 nm (reference: 655 nm) was measured in an iMark Microplate Reader (Bio-Rad). The antibody titer for a given sample was calculated as the reciprocal of the highest dilution of the test sample that gave an absorbance greater than a cutoff value equal to the mean + 3 standard deviation (SD) of 8 two-fold serial dilutions (starting at 1:20 and ending at 1:2560) of the negative control serum (uninfected mice).

### Surface plasmon resonance (SPR) assay

SPR assay was performed by using Biacore X100 (GE Healthcare Japan). Recombinant trimeric HA proteins of H2 JP305 with a C-terminal His-tag was immobilized on the surface of Sensor Chip NTA (GE Healthcare Japan) by using the NTA reagent kit (GE Healthcare Japan) according to the manufacturer’s instructions. After trimeric HA immobilization (1 μg/ml for 180 seconds), the molecular interaction of HA with either IgG or IgA was analyzed with a contact time of 60 seconds and a dissociation time of 600 seconds with an antibody concentration of either 100 μg/ml (Fig 3A) or 50μg/ml (Fig 3B and 3C). To compare the degree of antibody dissociation from recombinant HA proteins between multiple antibody forms, sensorgrams obtained from multiple analyses were x and y-axis adjusted (x = 0, y = 0: baseline, y = 100: binding).

### HI assay

HI titers were examined using a microtitration method as previously described, with minor modifications [42]. Briefly, purified antibodies (in duplicate) were serially diluted (two-fold), mixed with an equal volume of diluent containing influenza virus equivalent to 4 HA units, and incubated for 10 min at room temperature. Turkey red blood cells (RBCs) were added to the mixture, incubated at room temperature, and measurements were taken after 30 min. HI activity was defined as the reciprocal of the lowest concentration (μg/ml) of antibody that completely inhibited virus-mediated hemagglutination of RBCs.

### Neutralization (NT) assay

A microneutralization assay was performed using MDCK cells and 100 TCID_50_ (50% tissue culture infectious doses) of influenza virus essentially as previously described [43]. Briefly, 2-fold serial dilutions of each sample were mixed with an equal volume of diluent containing influenza virus equivalent to 100 TCID_50_. This was added to the wells of a 96-well plate containing a monolayer culture of MDCK cells. Four control wells containing virus or diluent alone were included on each plate. The plates were incubated for 5 days at 37°C/5% CO_2_. All wells were observed for the presence or absence of cytopathic effects by light microscopy. NT activity was defined as the reciprocal of the lowest concentration (μg/ml) of antibody that neutralized the virus.

### *In vivo* virus neutralization assay of recombinant IgG and SIgA antibodies

Mouse adapted A/Guizou/54/1989 (H3N2; Gui54) virus, which could infect mice and could be neutralized by F045-092 antibody was used for mice experiments. Virus was pre-incubated with either F045-092 IgG1, IgA1 monomer (A1Mo), IgA1 polymer (unfractionated crude mixture of IgA1 monomers and polymers; A1Poly), or PBS prior to mice challenge. 40 LD50 of Gui54 virus pre-incubated with F045-092 antibody (0.2 μg or 2 μg/head of either IgG1, A1Mo, or A1Poly) or PBS were intranasally administered to infect the upper (10 μl/head, 5 μl/nostril) and lower (20 μl/head) respiratory tracts of 7-week-old female BALB/c mice (six per experimental condition). Three days post-infection, nasal wash, and lung wash samples were collected and virus titers were measured by plaque assays. Mice were monitored daily for survival and weight loss until two weeks post-infection. Mice that lost 30% or more of their initial body weights were euthanized. Twenty-one days post-infection, serum samples were collected and anti-HA IgG titers were determined by ELISA.

### Plaque assays

Measurement of virus titers within mice nasal and lung wash samples were done by plaque assay, as described by Tobita et al. [44]. In brief, serial 10-fold dilutions of samples were prepared, and 200 μl aliquots of each dilution were inoculated into MDCK cells in a six-well plate. After 1 hr of incubation for sample absorption, each well was overlaid with 2 ml of agar medium. Plates were incubated for 2 days at 37°C/5% CO_2_ and stained with crystal violet for plaque counting. The virus titer of each experimental group was represented by the mean ± SD of pfu/ml of samples from six mice in each group.

### Statistical analysis

All statistical analyses were performed using the Prism statistical software package (version 6.0; GraphPad Software, Inc.). An unpaired Student t-test, the Holm-Sidak t-test, one-way ANOVA followed by Tukey’s multiple comparison test, the Kruskal-Wallis test followed by Dunn’s multiple comparison tests, or two-way ANOVA followed by Dunnett's multiple comparisons tests were used to analyze each data set as indicated in the figure legends. The threshold for statistical significance was set at 5% (*p* < 0.05). For statistical analyses of HI and NT titers, half of the detection limit value was applied to samples with titers below the detection limit.

## Supporting information

S1 Fig Reactivity of F045-092 bnAb against influenza A whole virions on IgA backbones with or without polymerization. (A) Reactivity of monomeric (red, A1Mo), dimeric (orange, A1Di), and tetrameric (blue, A1Te) F045-092 IgA1 antibodies against whole virions of A/New Caledonia/20/99 (H1N1; NC20) virus. Data are expressed as the mean ± SD of three technical replicates. An evident shift of reactivity curves to the right were observed by SIgA1 multimerization. (B) Area under the reactivity curve (AUC) for each IgA1 antibody (A1Mo, A1Di, and A1Te) against NC20 whole virions from the plots in (A). Data are expressed as the mean ± SD of three technical replicates. The reactivity of SIgA1 significantly increased by SIgA1 multimerization. (C) Reactivity of monomeric (red, A2m2Mo) and tetrameric (blue, A2m2Te) F045-092 IgA2m2 antibodies against whole virions of A/New Caledonia/20/99 (H1N1; NC20) virus. Data are expressed as the mean ± SD of three technical replicates. An evident shift of reactivity curves to the right were observed by SIgA2m2 tetramerization. (D) AUC for each IgA2m2 antibody (A2m2Mo, and A2m2Te) tested against NC20 whole virions. The AUC was calculated from the plots in (C). The reactivity of SIgA2m2 significantly increased by SIgA2m2 tetramerization. Data are expressed as the mean ± SD of three technical replicates. *p<0.05 (unpaired Student t-test or one-way ANOVA followed by Tukey’s multiple comparison test).
(TIF)

S2 Fig *In vivo* passive transfer experiment of recombinant IgG and SIgA antibodies in mice. (A) *In vitro* neutralizing activities of IgG1, monomeric (A1Mo), and polymeric (A1Poly) F045-092 IgA1 antibodies against A/Guizou/54/1989 (H3N2; Gui54) virus. NT activity is expressed on scatter plots as the geometric mean (with 95% confidence intervals) of the reciprocal of the lowest concentration (μg/ml) of antibody that neutralized the virus (n = 6 for each antibody). All antibody forms presented high NT activity against virus in vitro, and no significant difference could be observed between antibody forms. (B) Mice (six per experimental condition) were infected with Gui54 virus pre-incubated with F045-092 antibody (0.2 μg or 2 μg/head of either IgG1, A1Mo, or A1Poly) or PBS. The percentage of initial body weight and survival were plotted. Data are represented as mean ± SD. All mice administered with antibody pre-incubated virus survived. (C, D) Virus titers within lung (C) and nasal (D) wash samples collected on day 3 post infection. Virus titers are expressed on scatter plots as the mean ± SD. Virus titers could only be measured in the PBS group, and no infectious virus was detected from the groups infected with virus pre-incubated with antibodies. (E) Serum IgG antibody titers against H3 HA proteins in serum samples collected on day 21 post infection were measured by ELISA. Serum IgG responses against recombinant HA proteins from A/Beijing/353/1989 (H3N2; BJ353) virus was observed in one out of 6 mice in groups administered with virus pre-incubated with 0.2 μg IgG1 or A1Mo. This indicated the occurrence of asymptomatic infections of Gui54 virus in these mice, which suggested that only A1Poly was able to completely inactivate virus with an equal amount of antibody. The dotted line in the graph represents the detection limit (DL) of each experiment.
(TIF)
