# Supplementary figures and images for: IgA tetramerization improves target breadth but not peak potency of functionality of anti-influenza virus broadly neutralizing antibody
Source: PLoS Pathog. 2019 Jan 3;15(1):e1007427. doi: 10.1371/journal.ppat.1007427 (PMC6317788; doi:10.1371/journal.ppat.1007427)

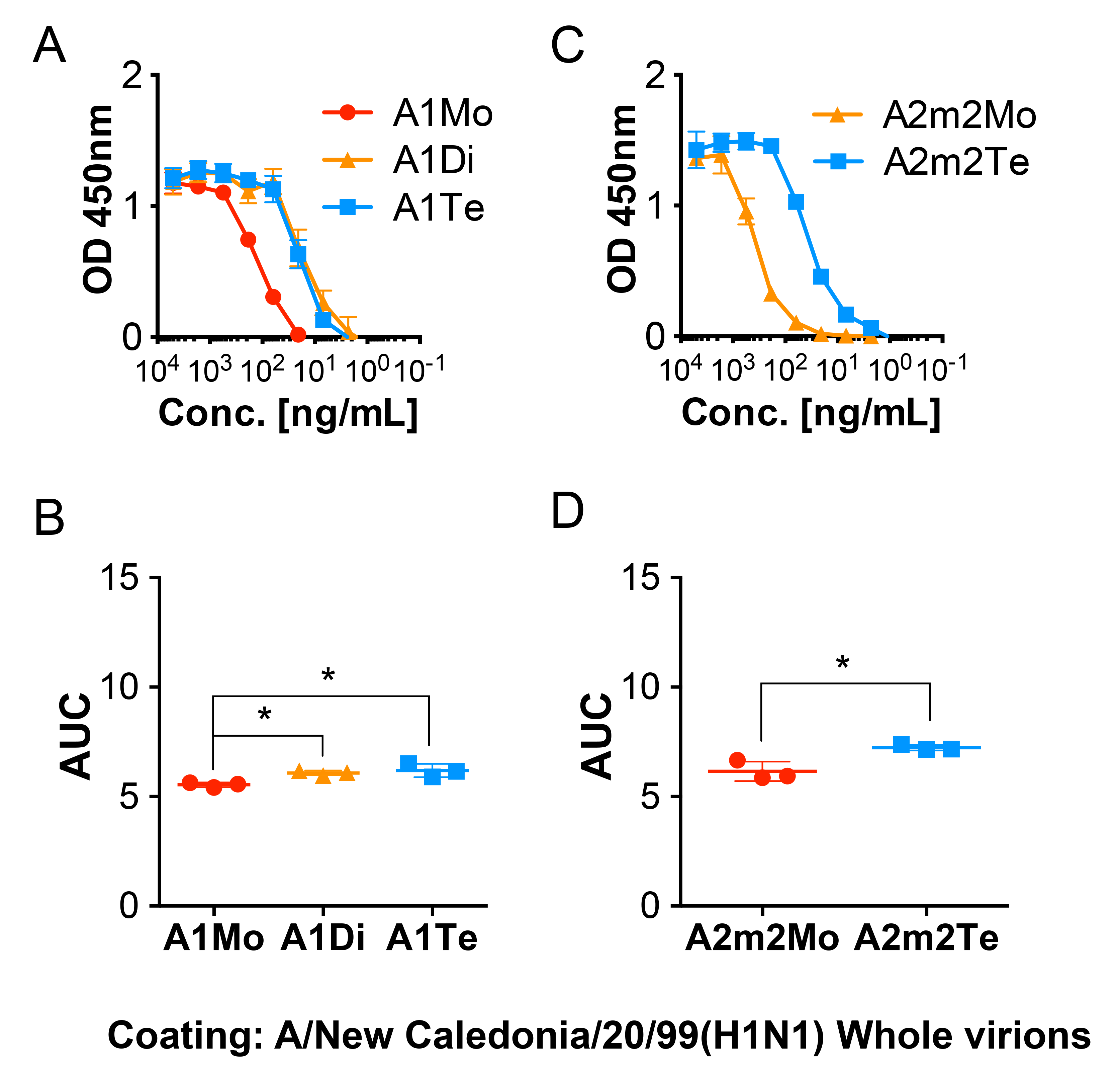

Supplement: S1 Fig — (A) Reactivity of monomeric (red, A1Mo), dimeric (orange, A1Di), and tetrameric (blue, A1Te) F045-092 IgA1 antibodies against whole virions of A/New Caledonia/20/99 (H1N1; NC20) virus. Data are expressed as the mean ± SD of three technical replicates. An evident shift of reactivity curves to the right were observed by SIgA1 multimerization. (B) Area under the reactivity curve (AUC) for each IgA1 antibody (A1Mo, A1Di, and A1Te) against NC20 whole virions from the plots in (A). Data are expressed as the mean ± SD of three technical replicates. The reactivity of SIgA1 significantly increased by SIgA1 multimerization. (C) Reactivity of monomeric (red, A2m2Mo) and tetrameric (blue, A2m2Te) F045-092 IgA2m2 antibodies against whole virions of A/New Caledonia/20/99 (H1N1; NC20) virus. Data are expressed as the mean ± SD of three technical replicates. An evident shift of reactivity curves to the right were observed by SIgA2m2 tetramerization. (D) AUC for each IgA2m2 antibody (A2m2Mo, and A2m2Te) tested against NC20 whole virions. The AUC was calculated from the plots in (C). The reactivity of SIgA2m2 significantly increased by SIgA2m2 tetramerization. Data are expressed as the mean ± SD of three technical replicates. *p<0.05 (unpaired Student t-test or one-way ANOVA followed by Tukey’s multiple comparison test). (TIF) [file ppat.1007427.s001.tif]

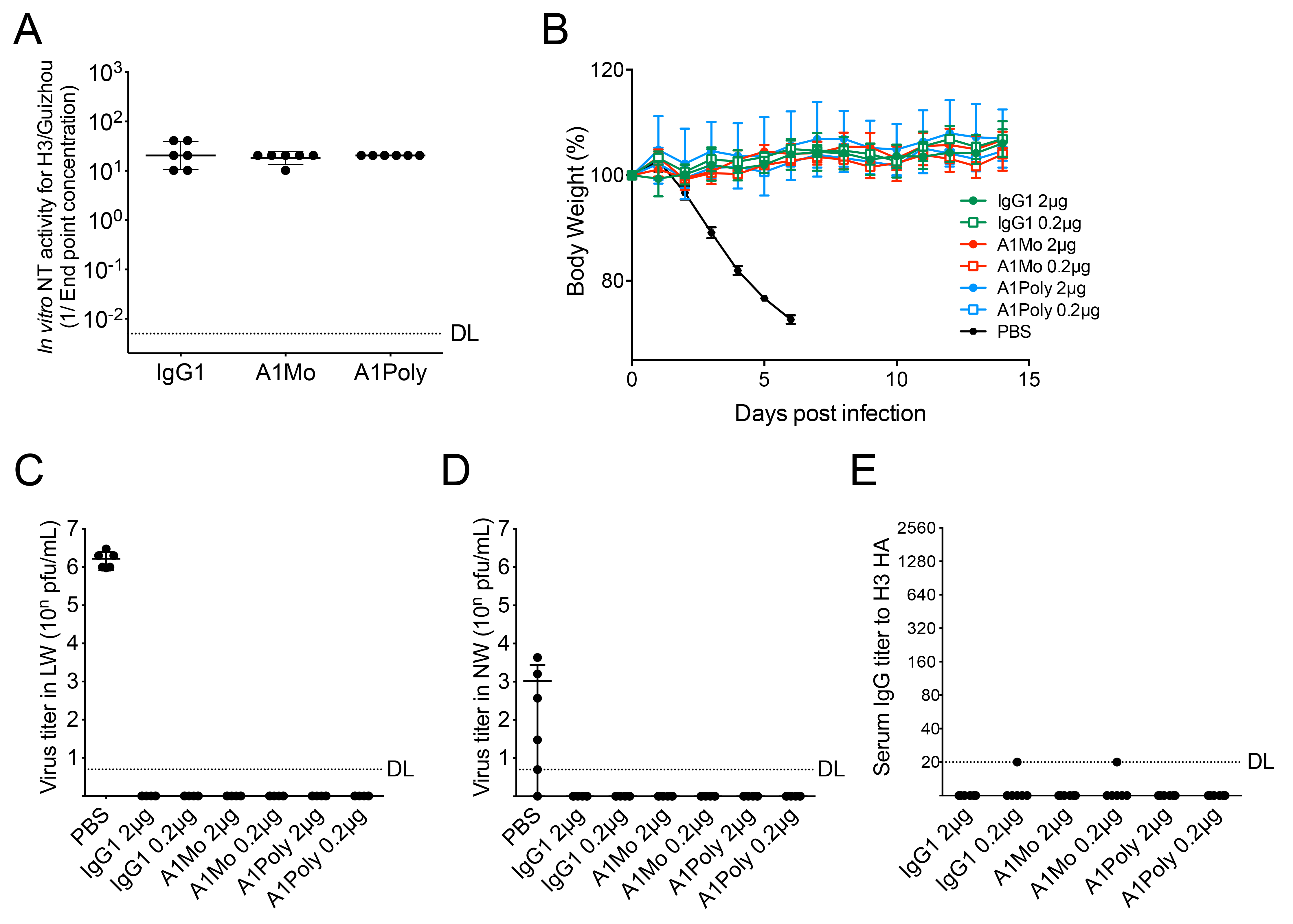

Supplement: S2 Fig — (A) In vitro neutralizing activities of IgG1, monomeric (A1Mo), and polymeric (A1Poly) F045-092 IgA1 antibodies against A/Guizou/54/1989 (H3N2; Gui54) virus. NT activity is expressed on scatter plots as the geometric mean (with 95% confidence intervals) of the reciprocal of the lowest concentration (μg/ml) of antibody that neutralized the virus (n = 6 for each antibody). All antibody forms presented high NT activity against virus in vitro, and no significant difference could be observed between antibody forms. (B) Mice (six per experimental condition) were infected with Gui54 virus pre-incubated with F045-092 antibody (0.2 μg or 2 μg/head of either IgG1, A1Mo, or A1Poly) or PBS. The percentage of initial body weight and survival were plotted. Data are represented as mean ± SD. All mice administered with antibody pre-incubated virus survived. (C, D) Virus titers within lung (C) and nasal (D) wash samples collected on day 3 post infection. Virus titers are expressed on scatter plots as the mean ± SD. Virus titers could only be measured in the PBS group, and no infectious virus was detected from the groups infected with virus pre-incubated with antibodies. (E) Serum IgG antibody titers against H3 HA proteins in serum samples collected on day 21 post infection were measured by ELISA. Serum IgG responses against recombinant HA proteins from A/Beijing/353/1989 (H3N2; BJ353) virus was observed in one out of 6 mice in groups administered with virus pre-incubated with 0.2 μg IgG1 or A1Mo. This indicated the occurrence of asymptomatic infections of Gui54 virus in these mice, which suggested that only A1Poly was able to completely inactivate virus with an equal amount of antibody. The dotted line in the graph represents the detection limit (DL) of each experiment. (TIF) [file ppat.1007427.s002.tif]
